# Attenuation of Carcinogenesis and the Mechanism Underlying by the Influence of Indole-3-carbinol and Its Metabolite 3,3′-Diindolylmethane: A Therapeutic Marvel

**DOI:** 10.1155/2014/832161

**Published:** 2014-05-08

**Authors:** V. L. Maruthanila, J. Poornima, S. Mirunalini

**Affiliations:** Department of Biochemistry and Biotechnology, Faculty of Science, Annamalai University, Annamalainagar, Tamilnadu 608 002, India

## Abstract

Rising evidence provides credible support towards the potential role of bioactive products derived from cruciferous vegetables such as broccoli, cauliflower, kale, cabbage, brussels sprouts, turnips, kohlrabi, bok choy, and radishes. Many epidemiological studies point out that *Brassica* vegetable protects humans against cancer since they are rich sources of glucosinolates in addition to possessing a high content of flavonoids, vitamins, and mineral nutrients. Indole-3-carbinol (I3C) belongs to the class of compounds called indole glucosinolate, obtained from cruciferous vegetables, and is well-known for tits anticancer properties. In particular, I3C and its dimeric product, 3,3′-diindolylmethane (DIM), have been generally investigated for their value against a number of human cancers *in vitro* as well as *in vivo*. This paper reviews an in-depth study of the anticancer activity and the miscellaneous mechanisms underlying the anticarcinogenicity thereby broadening its therapeutic marvel.

## 1. Introduction

Plant materials present in the human diet contain a large number of naturally occurring compounds that may be useful to protect the body against cancer development. Recently, many candidates have been assayed to identify the presence of anticarcinogens in their diet [1]. Recognition of diet as a primary causative factor to overcome cancer risk has directed much research attention towards the chemoprotective role of certain compounds in foods [2]. Technological progress in manipulating plant metabolism and metabolites, combined with the explosive growth of the “functional food” industry, has led to many attempts to enhance the concentration of these health-promoting compounds in specific plant-based foods [2].

The anticancer properties of cruciferous vegetables are primarily documented by the Roman statesman, Cato and Elder (234-149 BC), who in his study of drug wrote “if a cancerous ulcer appears on the breasts, apply a crushed cabbage leaf and it will make it well [3].” It is now well set up that cruciferous vegetables contain a forerunner phytochemical openly glucosinolate that undergoes hydrolysis by the plant enzyme myrosinase, yielding a bioactive compound known as I3C [3]. It is rapidly converted to many condensation products as it is chemically unbalanced in aqueous and gastric acidic environment. The dietary indoles, I3C and DIM, occur naturally as glucosinolate conjugates in* Brassica* vegetables and are released upon hydrolysis [3]. The cancer-protecting properties of* Brassica *(i.e., broccoli, cauliflower, kale, cabbage, brussels sprouts, bok choy, radishes, turnips, and kohlrabi) utilization are most likely mediated through “bioactive compounds” that induce a variety of physiological process including direct or indirect antioxidant action, detoxifying enzymes, inducing apoptosis, and cell cycle regulation [4].

DIM is readily detected in the livers and feces of rodents fed I3C, whereas the parent I3C compound has not been detected in tissues of these rodents [5]. Thus, the natural effects of I3C are attributable to DIM, which show evidence of antitumorigenic activities* in vivo* and* in vitro* by reducing the growth of prostate, colon, and breast cancer cells [6, 7]. I3C and its derivatives also suppress cell propagation and induce apoptosis in colon cancer cells [8], as well as in other types of cancer cells including prostate [9], breast [7], bladder [10], pancreas [11], and hepatoma [12]. This review mainly focuses on the role of I3C and DIM against various types of malignancy and underlying mechanisms.

## 2. Cruciferous Vegetables and Their Derivatives

Glucosinolates are a class of organic compounds that give pungent smell and piquant taste in the cruciferous vegetables and some condiments, such as wasabi and mustard. The main function of glucosinolates in plants is that it acts as natural pesticides and accelerates the resistance against herbivores [13].

**Figure 1:**
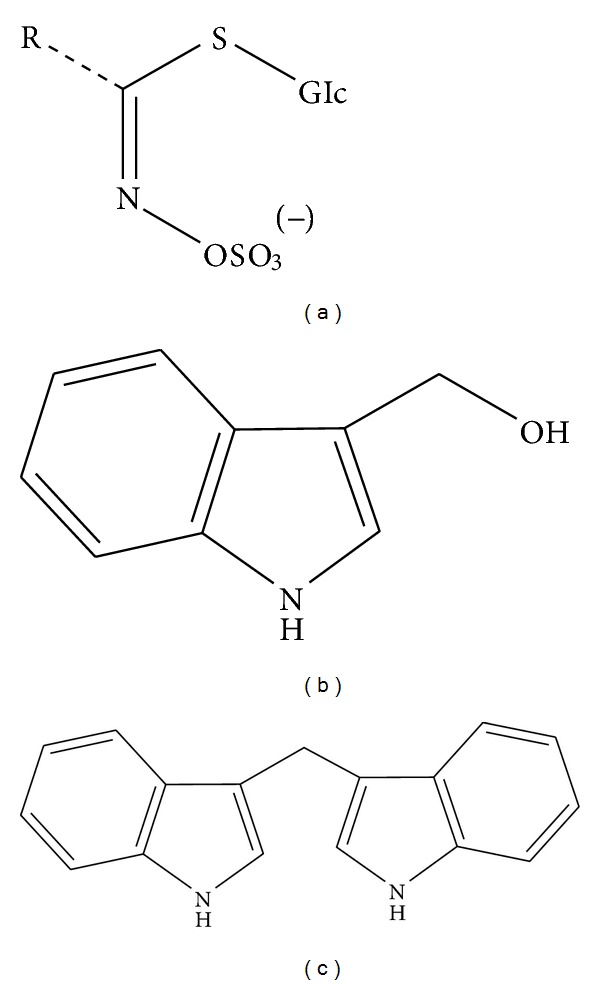
Structures of (a) glucosinolates, (b) I3C, and (c) DIM.

The central carbon of all glucosinolates is bound 6 to a thioglucose group to a sulfate group via the nitrogen molecule [22]. The central carbon of each glucosinolate is bound to a side group and this makes each glucosinolate unique [22]. Glucosinolates are classified as aliphatic (e.g., alkenyl, alkyl, hydroxyalkenyl, or w-methylthioalkyl), aromatic (e.g., benzyl, substituted benzyl), or heterocyclic with the R-group of the aliphatic glucosinolates being derived from alanine, methionine, leucine, or valine and those of aromatic and heterocyclic glucosinolates being derived from phenylalanine or tyrosine or tryptophan** [**14] (Figure 1(a)). Although glucosinolates are related to inhibition of carcinogenesis, it is actually their hydrolysis products, not the glucosinolates themselves that are biologically active. Hydrolysis of glucosinolates is catalyzed by the enzyme myrosinase, also known as *β*-thioglucoside glucohydrolase [13]. In a low pH environment, I3C is converted into polymeric products and DIM is the main one. DIM is a major* in vivo *acid catalyzed condensation product of I3C [23] (Figures 1(b) and 1(c)). They are no more natural products from DIM. Maciejewska et al. synthesized a series of DIM derivatives bearing fluoro, bromo, iodo, and nitro substituents in indole or benzene rings and tested for cytotoxicity against human melanoma cell lines after characterizing their structure [23]. These derivatives were found to cause 50% inhibition of the viability of ME18 and ME18/R cell lines at concentrations ranging between 9.7 and 17.3 mM [23]. These finding clearly suggested that synthetic analog of DIM has potential role in cancer therapy in the near future once their systemic toxicity studies have been determined [23].

## 3. Bioavailability of I3C and DIM

Reed et al. [24] in his extensive article of pharmacokinetic studies reported that women received oral doses of 400, 600, 800, 1,000, and 1,200 mg I3C and these serial plasma samples were analyzed by high-performance liquid chromatography-mass spectrometry method for the detection and quantitation of the I3C and DIM. I3C itself is not detectable in plasma [24]. The only detectable I3C-derived product is DIM. High initial value, plasma DIM for all subjects, decreased to near or below the limit of quantitation within the 12 h sampling period [24]. Physiologically based pharmacokinetic (PBPK) model is developed using plasma and tissue (brain, heart, liver, kidneys, and lungs) concentration data for DIM to compare the pharmacokinetic properties and biodistribution of pure crystalline and a novel formulation (BioResponse-DIM (BR-DIM)) of DIM after oral administration to mice [25]. I3C is hard to develop as a drug because it is highly unstable and can transform into many other derivatives such as DIM, 5,6,11,12,17,18-hexahydrocyclonona[1,2-b:4,5-b′:7,8-b′′] triindole (CTr), indolo [3,2-b] carbazole (ICZ), N-methoxyindole-3-carbinol (NI3C), two tetramers, one linear (LTET) and one cyclic (CTET), and 1-(3-hydroxymethyl) indolylmethane (HI-IM). Anderton et al. reported oral doses of I3C to female CD-1 mice, and studied the disposition of I3C and its acid condensation products DIM, ICZ, LTr(1), and HI-IM in blood, liver, kidney, lung, heart, and brain. I3C was rapidly absorbed, distributed, and eliminated from plasma and tissues, falling below the limit of detection by 1 h [5]. The major acid condensation product of I3C, DIM, has proven very stable in acidic conditions during prolonged exposure to high temperature and humidity [4]. Another report BR-DIM has proven very stable and it is not converted into other forms [4]. Based on these outcomes authors suggested that one could exploit further development of DIM as a potential therapeutic agent.

## 4. Metabolism and Distribution

Under the acidic conditions of the gastric tract, I3C undergoes condensation to form several oligomeric products, particularly DIM [23]. The tissue distribution of I3C has been determined in mice by radio-labelled I3C [26]. Both I3C and DIM have been detected in kidneys, lungs, heart, liver, plasma, and brain samples of mice treated with 250 mg/kg of I3C as early as 15 mins after administration [26]. These results suggest that I3C is rapidly absorbed and spread to a number of well-perfused tissues, where it is transformed to DIM to perform its anticancer actions [26].

## 5. Epidemiological Studies

For more than 25 years, the interdependence between nutrition and the development and progression of cancer have been recognized [27]. The key challenge is to identify the specific components responsible for contributing to this relationship [27]. In the United States, cancers of the lungs, colon, rectum, breast, and prostate account for almost half of the total cancer incidence [27]. Cohen et al. [28] examined the association of fruit and vegetable intake and prostate cancer risk among newly diagnosed men residing in the Seattle, WA, area. With each 10 g of cruciferous vegetables consumed per day, one could expect an 8% decrease in risk for colorectal cancer.

Several recent case-control studies in the US, Sweden, and China found that measures of cruciferous vegetable intake are significantly lower in women diagnosed with breast cancer than in cancer-free control groups [29]. High intake of cruciferous vegetables has been associated with lower risk of lung and colorectal cancer in some epidemiological studies, but there is evidence that genetic polymorphisms may influence the effectiveness of cruciferous vegetables on human cancer risk [30]. Epidemiological studies show that consumption of large quantities of fruits and vegetables, particularly cruciferous vegetables, is associated with a reduced occurrence of cancer [30].

## 6. Experimental Studies

Studies in various experimental models have shown that I3C can alter the metabolism of carcinogens and provide protection against chemically induced carcinogenesis [31]. When administered before or at the same time as the carcinogen, oral I3C has been originated to inhibit the spreading out of cancer in a variety of animal models and tissues, including cancers of the mammary gland (breast) [7], colon [8], stomach [31], lung [26], and liver [32]). However, a number of studies have found that I3C actually promoted or enhanced the development of cancer when administered chronically after the carcinogen [32].

The cancer promoting effects of I3C is first reported in a trout model of liver cancer [32]. However, I3C also has been found to promote cancer of the thyroid, liver, colon, and uterus in rats [33]. Although the long-term effects of I3C supplementation on cancer risk in humans are not known, but the contradictory results of animal studies have led some to caution against the widespread use of I3C and DIM supplements in humans until their potential risks and benefits are better understood [33].

## 7. Therapeutic Action of Indole Glucosinolates

### 7.1. Apoptosis

**Figure 2:**
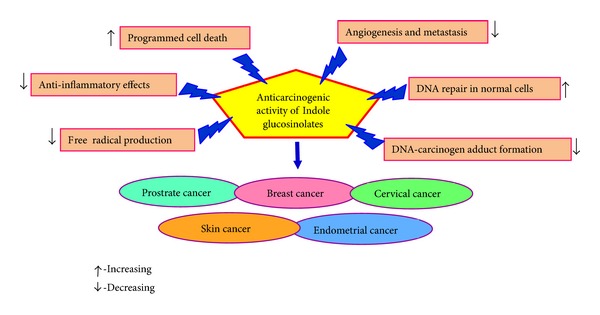
Sources of anticancer effect of indole glucosinolates (I3C and DIM) and its mode of action.

Apoptosis or programmed cell death is a highly regulated process that involves activation of a series of molecular events, leading to cell death that is characterized by cellular morphological change, chromatin condensation, and apoptotic bodies which are associated with DNA cleavage into ladders [34]. The nuclear factor kappa B (NF-*κ*B) signaling plays critical roles in regulating cell proliferation, survival, tumor invasion, metastasis, drug resistance, and stress response [35]. We confirmed that NF-*κ*B activity is significantly upregulated by docetaxel, gemcitabine or oxaliplatin treatment and that the NF-*κ*B inducing activity of these agents was completely abrogated in cells pretreated with DIM [36]. We found that DIM, or the formulated BR-DIM treatment, could restrict its nuclear localization and inactivate NF-*κ*B DNA-binding activity in prostate [14], breast [15], and pancreatic cancer cells [36], resulting in the inhibition of transcriptional downregulation of several NF-*κ*B downstream genes causing inhibition of cell growth and inducing apoptotic cell death. Collectively, these results clearly suggest that DIM pretreatment, which inactivates NF-*κ*B activity, along with other cellular effects of DIM, may contribute to enhanced cell growth inhibition and apoptosis with suboptimal doses of cytotoxic chemotherapeutic agents with minimal side effects (Figure 2).

I3C trigger the stress-induced MAP-kinases p38 and C-jun N-terminal kinase (JNK) in prostate cancer cells and to inhibit constitutively active STAT3, a transcription factor, in pancreatic cancer cells [18]. Irrespective of the cell type I3C suppressed NF-*κ*B activation induced by various agents [18]. NF-*κ*B inhibition correlated with suppression of inhibitor of kappa B kinase (IKK) and I*κ*B*α* phosphorylation, ubiquitination, degradation with p65 phosphorylation, nuclear translocation, and acetylation [20]. I3C also downregulated NF-*κ*B *α* regulated reporter gene transcription and gene products involved in cell proliferation, antiapoptosis, and invasion [20]. This led to the potentiation of apoptosis induced by cytokines and chemotherapeutic agents [20]. Collectively, the concerted effects on those proapoptotic components underlie the ability of I3C/DIM to induce mitochondrial dependent apoptosis in tumor cells [20].

### 7.2. Regulation of Redox Status

Reactive oxygen species (ROS) including H_2_O_2_ can cause different combinations of apoptosis, necrosis, and autophagy in a cell line dependent and stimulus-dependent manner [37]. The capacity of I3C to form adducts with electrophiles or free radicals appears too autonomous on their chemical reactivity hence the scavenging ability of I3C is compatible with the adduct formation [38]. Arnao et al. [38] investigate the ability of I3C to trap a metastable synthetic-free radical and inhibition of carcinogenesis. This induction may be produced by I3C itself and/or I3C derived polymerization products such as DIM and others [39].

According to Benabadji et al. [40], they reported that DIM and 6-methoxy-DIM in DPPH model, their IC_50_, were 50% and 40% smaller than that of vitamin E, due to their hydrogen-donating ability with the presence of two N–H groups as an H-donating group necessary to react with free radical and slightly less potent than the standard phenolic antioxidant BHA in *β*-carotene model with IC_50_ 4% and 9% smaller for DIM and 6-methoxy-DIM.

### 7.3. Anti-Inflammatory Effect

The effect of DIM on inflammatory responses and its molecular mechanisms of DIM have been examined using lipopolysaccharide (LPS) stimulated RAW264.7 murine macrophages [16]. DIM inhibits LPS-induced increases in protein levels of inducible nitric oxide synthase (iNOS), which are accompanied by decreased iNOS mRNA levels and transcriptional activity [16]. In addition, DIM suppresses LPS-induced NF-*κ*B transcriptional and DNA-binding activity, translocation of p65 (RelA) to the nucleus, and degradation of inhibitor of kappa B alpha (I*κ*B*α*) [16]. Also the results of RT-PCR analysis exposed that LPS augmented the steady-state levels of proinflammatory cytokines such as TNF-*α*, IL-1*β*, PLA2, and interleukin-6 (IL-6) transcripts, which are substantially suppressed by DIM treatment [16]. DIM pretreatment significantly repressed LPS-induced phosphorylation of SAPK/JNK, whereas the phosphorylation of other MAPK family proteins (p38 or ERK-1/2) is unaltered by DIM pretreatment [41].

### 7.4. Cell Cycle Arrest

Cell cycle arrest is defined as the halt of the cell cycle. I3C is reported to inhibit cyclin dependent kinase 2 (CDK2) kinase activities in MCF-7 cells through selective alterations in cyclin E composition, size distribution, and subcellular localization of the CDK2 protein complex [42]. Cell-cycle arrest involves the upregulation of the CDK inhibitors p21 WAF1 and p27 KIP1 and the concurrent downregulation of cyclin D1, cyclin E, and CDKs 2, 4, and 6 attributable to the effect of I3C and DIM on regulating SP1-promoter binding activity [19]. Inhibition of CDK4/6 cyclin D1 and CDK2 cyclin E activities led to decreased Rb phosphorylations, which cause the Rb protein to bind to the E2F transcription factor [43]. This E2F sequestration blocks the transcription of S phase genes resulting in G_1_ arrest; also the involvement of p53 and cell cycle arrest in the I3C-mediated effect has been studied in various cancer cells [44].

Treatment of I3C betters the expression of p53 (Ser 15) and CDKIs such as p21 and p27, while cyclin D1 expression is suppressed and cyclin E is not altered [45]. Collectively, the data for the cell cycle analysis, the Plk-1 assay, and the western blot of p53 and CDKIs explain that I3C augments the expression of p53 and CDKIs and that I3C induced cell cycle arrest at G_0_/G_1_ in A549 cells [45]. In addition, cotreatment with I3C and wortmannin prevents both phosphorylation of p53 at Ser 15 and p21 expression. Hence it is clear that A549 cell arrest by I3C is involved in the PI3 K and p53 signal pathways [45].

### 7.5. Angiogenesis

Angiogenesis is the physiological process through which new blood vessels form from preexisting vessels. This is distinct from vasculogenesis, which is the* de novo* formation of endothelial cells from mesoderm cell precursors [46]. Dysregulated angiogenesis that consists of the unbalanced production of pro- and antiangiogenic factors is linked to a number of pathological situations [21]. For example, the overexpression of angiogenic factors, including vascular endothelial growth factor (VEGF), IL-6, and matrix metalloproteinases (MMP-9) is closely associated with the development of cancers and metastasis [21]. The effect of I3C on LPS-activated macrophage-induced tube formation and its associated factors in endothelial EAhy926 cells are investigated [21]. LPS significantly enhanced the capillary-like structure of endothelial cells (ECs) cocultured with macrophages, but no such effect was observed in single-cultured ECs [21]. I3C, on the other hand, suppressed such enhancement in concert with decreased secretions of VEGF, NO, IL-6, and MMPs [21]. The results obtained from cultivating ECs with conditioned medium (CM) collected from macrophages suggested that both ECs and macrophages were inactivated by I3C [21].

### 7.6. Detoxification

Detoxification is the physiological or medicinal removal of toxic substances from living organisms [47]. Both the Phase I and Phase II detoxification centers in the liver and the intestinal epithelial cells can be accelerated by some minor exogenous agent like I3C [47]. Many researchers indicate that the ability of cruciferous vegetables to motivate Phase I and Phase II detoxification, particularly their I3C content, is a primary factor in which these nutrients are related to reduce cancer risk in humans [48]. Animals are exposed to or injected with carcinogens; the animals receiving the cruciferous vegetables or the I3C in their food supply have a significantly lower tumor incidence than the animals fed the same diet, but without cruciferous vegetables or I3C fortification [48].  Table 1 showed the genes modulated by I3C and DIM in the above mechanisms.

## 8. Anticancer Effects of I3C and DIM

DIM inhibits proliferation of human breast cancer cells at concentrations achievable through oral supplementation with I3C (10–50 *μ*M) [49]. Recently a report by Fan et al. [50] demonstrated that DIM protects cancer cells and normal epithelial cells against ROS in a breast cancer type 1 susceptibility protein (BRCA1-)dependent manner. Moreover I3C inhibits DMBA initiated and TPA promoted mouse skin tumour formation [51]. I3C also exhibits inhibitory and preventive effects on prostate tumors in mice [9]. Other investigators reported that this activity of I3C is associated with its action as a nonspecific inducer of powerful cytochrome enzymes responsible for “Phase I” detoxification metabolism, the efficiency of I3C to inhibit prostate tumor growth [52]. The anticancer efficacy of I3C is well proven including the reduction of cervical intraepithelial neoplasia (CIN) and its progression to cervical cancer [53]. It is perceived that the diminished phosphatase and tensin homolog protein (PTEN) expression is observed during the progression from low-grade to high-grade cervical dysplasia in humans and in a mouse model for cervical cancer, the K14HPV16 transgenic mice promoted with estrogen [53]. PTEN could impede tumor progression by inhibiting proliferation and by increasing tumor cell apoptosis [53]. This is supported by the previous findings that I3C decreased proliferating cell nuclear antigen- (PCNA-)positive cells and increased TdT-mediated dUTP nick-end labeling- (TUNEL-)positive cells in abnormal cervical epithelium of HPV16 mice [54].

## 9. Drug Resistance

While a number of therapeutic options are available for the cure of various cancers, a major clinical problem is the development of drug resistance [55]. Intrinsic and acquired resistances are the two broad classifications of resistance to anticancer drugs. Natural compounds, such as indoles, which induce apoptosis in human cancer cells without causing unwanted toxicity in normal cells, can be useful in combination with conventional chemotherapeutic agents for the treatment of human malignancies with diminished toxicity and higher efficacy [55].

## 10. Conclusion

As seen throughout the review, cruciferous vegetables impart several mechanisms of action to develop its outsized number of function. The experimental studies as described above suggest that I3C and DIM, the components of cruciferous vegetables, have therapeutic potential both for prevention and for treatment of cancer. Moreover the future research can focus on the advantageous effect of I3C and DIM in various clinical studies to overcome the cancer epidemic among the population by the application of diverse technologies.

## Figures and Tables

**Table 1:** Modulation of genes involved in various mechanisms by the influence of I3C and DIM.

Mechanisms involved	Upregulated genes (↑) modulated by I3C and DIM	Downregulated genes (↓) modulated by I3C and DIM	References
Apoptosis	JNK/SAPK and Bax	Bcl-xl, Bcl-2, surviving, and NF-*κ*B	[14–16]
Xenobiotic metabolism	CYP, CYP 1A1, CYP 1A2, and CYP 1B1	—	[17]
Antioxidant	GSH and GST	—	[4]
Transcription factor	Nrf2 and ATF3	NF-*κ*B and STAT3	[4, 18]
Cell cycle	p21 WAF1, and p27 KIP1	Cyclin D1, E, CDK2, and CDK6	[19]
Inflammation	NAG-1	NF-*κ*B and MMP-9	[16, 20]
Angiogenesis	VEGF, IL-6, and MMP-9	—	[21]

## References

[1] Wattenberg LW (1990). Inhibition of carcinogenesis by minor anutrient constituents of the diet. *Proceedings of the Nutrition Society*.

[2] Finley JW (2005). Proposed criteria for assessing the efficacy of cancer reduction by plant foods enriched in carotenoids, glucosinolates, polyphenols and selenocompounds. *Annals of Botany*.

[3] de Kruif CA, Marsman JW, Venekamp JC (1991). Structure elucidation of acid reaction products of indole-3-carbinol: detection *in vivo* and enzyme induction *in vitro*. *Chemico-Biological Interactions*.

[4] Banerjee S, Kong D, Wang Z, Bao B, Hillman GG, Sarkar FH (2011). Attenuation of multi-targeted proliferation-linked signaling by 3,3′-diindolylmethane (DIM): from bench to clinic. *Mutation Research*.

[5] Anderton MJ, Manson MM, Verschoyle RD (2004). Pharmacokinetics and tissue disposition of indole-3-carbinol and its acid condensation products after oral administration to mice. *Clinical Cancer Research*.

[6] Kim DJ, Shin DH, Ahn B (2003). Chemoprevention of colon cancer by Korean food plant components. *Mutation Research*.

[7] Rahman KMW, Sarkar FH (2005). Inhibition of nuclear translocation of nuclear factor-*κ*B contributes to 3,3′-Diindolylmethane-induced apoptosis in breast cancer cells. *Cancer Research*.

[8] Chintharlapalli S, Smith R, Samudio I, Zhang W, Safe S (2004). 1,1-Bis(3′-indolyl)-1-(p-substitutedphenyl)methanes induce peroxisome proliferator-activated receptor *γ*-mediated growth inhibition, transactivation, and differeatiation markers in colon cancer cells. *Cancer Research*.

[9] Nachshon-Kedmi M, Yannai S, Haj A, Fares FA (2003). Indole-3-carbinol and 3,3′-diindolylmethane induce apoptosis in human prostate cancer cells. *Food and Chemical Toxicology*.

[10] Kassouf W, Chintharlapalli S, Abdelrahim M, Nelkin G, Safe S, Kamat AM (2006). Inhibition of bladder tumor growth by 1,1-bis(3′-indolyl)-1-(p- substitutedphenyl)methanes: a new class of peroxisome proliferator-activated receptor *γ* agonists. *Cancer Research*.

[11] Abdelrahim M, Newman K, Vanderlaag K, Samudio I, Safe S (2006). 3,3′-Diindolylmethane (DIM) and its derivatives induce apoptosis in pancreatic cancer cells through endoplasmic reticulum stress-dependent upregulation of DR5. *Carcinogenesis*.

[12] Gong Y, Firestone GL, Bjeldanes LF (2006). 3,3′-Diindolylmethane is a novel topoisomerase II*α* catalytic inhibitor that induces S-phase retardation and mitotic delay in human hepatoma HepG2 cells. *Molecular Pharmacology*.

[13] Keck A-S, Finley JW (2004). Cruciferous vegetables: cancer protective mechanisms of glucosinolate hydrolysis products and selenium. *Integrative Cancer Therapies*.

[14] Li Y, Chinni SR, Sarkar FH (2005). Selective growth regulatory and pro-apoptotic effects of DIM is mediated by Akt and NF-kappaB pathways in prostate cancer cells. *Frontiers in Bioscience*.

[15] Rahman KMW, Banerjee S, Ali S (2009). 3,3′-diindolylmethane enhances taxotere-induced apoptosis in hormone-refractory prostate cancer cells through survivin down-regulation. *Cancer Research*.

[16] Cho HJ, Seon MR, Lee YM (2008). 3,3′-Diindolylmethane suppresses the inflammatory response to lipopolysaccharide in murine macrophages. *The Journal of Nutrition*.

[17] Yoshida M, Katashima S, Ando J (2004). Dietary indole-3-carbinol promotes endometrial adenocarcinoma development in rats initiated with N-ethyl-N′ -nitro-N-nitrosoguanidine, with induction of cytochrome P450s in the liver and consequent modulation of estrogen metabolism. *Carcinogenesis*.

[18] Lian JP, Word B, Taylor S, Hammons GJ, Lyn-Cook BD (2004). Modulation of the constitutive activated STAT3 transcription factor in pancreatic cancer prevention effects of indole-3-carbinol (I3C) and genistein. *Anticancer Research*.

[19] Hong C, Firestone GL, Bjeldanes LF (2002). Bcl-2 family-mediated apoptotic effects of 3,3′-diindolylmethane (DIM) in human breast cancer cells. *Biochemical Pharmacology*.

[20] Takada Y, Andreeff M, Aggarwal BB (2005). Indole-3-carbinol suppresses NF-*κ*B and I*κ*B*α* kinase activation, causing inhibition of expression of NF-*κ*B-regulated antiapoptotic and metastatic gene products and enhancement of apoptosis in myeloid and leukemia cells. *Blood*.

[21] Kunimasa K, Kobayashi T, Kaji K, Ohta T (2010). Antiangiogenic effects of indole-3-carbinol and 3,3′-diindolylmethane are associated with their differential regulation of ERK1/2 and Akt in tube-forming HUVEC. *The Journal of Nutrition*.

[22] Hayes JD, Kelleher MO, Eggleston IM (2008). The cancer chemopreventive actions of phytochemicals derived from glucosinolates. *European Journal of Nutrition*.

[23] Maciejewska D, Rasztawicka M, Wolska I, Anuszewska E, Gruber B (2009). Novel 3,3′-diindolylmethane derivatives: Synthesis and cytotoxicity, structural characterization in solid state. *European Journal of Medicinal Chemistry*.

[24] Reed GA, Arneson DW, Putnam WC (2006). Single-dose and multiple-dose administration of indole-3-carbinol to women: pharmacokinetics based on 3,3′-diindolylmethane. *Cancer Epidemiology Biomarkers and Prevention*.

[25] Anderton MJ, Manson MM, Verschoyle R (2004). Physiological modeling of formulated and crystalline 3,3′ -diindolylmethane pharmacokinetics following oral administration in mice. *Drug Metabolism and Disposition*.

[26] Kassie F, Anderson LB, Scherber R (2007). Indole-3-carbinol inhibits 4-(methylnitrosamino)-1-(3-pyridyl)-1-butanone plus benzo(a)pyrene-induced lung tumorigenesis in A/J mice and modulates carcinogen-induced alterations in protein levels. *Cancer Research*.

[27] Murillo G, Mehta RG (2001). Cruciferous vegetables and cancer prevention. *Nutrition and Cancer*.

[28] Cohen JH, Kristal AR, Stanford JL (2000). Fruit and vegetable intakes and prostate cancer risk. *Journal of the National Cancer Institute*.

[29] Ambrosone CB, McCann SE, Freudenheim JL, Marshall JR, Zhang Y, Shields PG (2004). Breast cancer risk in premenopausal women is inversely associated with consumption of broccoli, a source of isothiocyanates, but is not modified by GST genotype. *The Journal of Nutrition*.

[30] Higdon JV, Delage B, Williams DE, Dashwood RH (2007). Cruciferous vegetables and human cancer risk: epidemiologic evidence and mechanistic basis. *Pharmacological Research*.

[31] Wattenberg LW, Loub WD (1978). Inhibition of polycyclic aromatic hydrocarbon-induced neoplasia by naturally occurring indoles. *Cancer Research*.

[32] Hendrich S, Bjeldanes LF (1983). Effects of dietary cabbage, Brussels sprouts, Illicium verum, Schizandra chinensis and alfalfa on the benzo[a]pyrene metabolic system in mouse liver. *Food and Chemical Toxicology*.

[33] Lee BM, Park K-K (2003). Beneficial and adverse effects of chemopreventive agents. *Mutation Research*.

[34] Chen Y-C, Lin-Shiau S-Y, Lin J-K (1999). Involvement of p53 and HSP70 proteins in attenuation of UVC-induced apoptosis by thermal stress in hepatocellular carcinoma cells. *Photochemistry and Photobiology*.

[35] Karin M, Greten FR (2005). NF-*κ*B: linking inflammation and immunity to cancer development and progression. *Nature Reviews, Immunology Cancer Cell*.

[36] Banerjee S, Wang Z, Kong D, Sarkar FH (2009). 3,3′-diindolylmethane enhances chemosensitivity of multiple chemotherapeutic agents in pancreatic cancer. *Cancer Research*.

[37] Chen Y, McMillan-Ward E, Kong J, Israels SJ, Gibson SB (2008). Oxidative stress induces autophagic cell death independent of apoptosis in transformed and cancer cells. *Cell Death and Differentiation*.

[38] Arnao MB, Sanchez-Bravo J, Acosta M (1996). Indole-3-carbinol as a scavenger of free radicals. *Biochemistry and Molecular Biology International*.

[39] Sun S, Han J, Ralph WM (2004). Endoplasmic reticulum stress as a correlate of cytotoxicity in human tumor cells exposed to diindolylmethane *in vitro*. *Cell Stress and Chaperones*.

[40] Benabadji SH, Wen R, Zheng J-B, Dong X-C, Yuan S-G (2004). Anticarcinogenic and antioxidant activity of diindolylmethane derivatives. *Acta Pharmacologica Sinica*.

[41] Karin M (2005). Inflammation-activated protein kinases as targets for drug development. *Proceedings of the American Thoracic Society*.

[42] Garcia HH, Brar GA, Nguyen DHH, Bjeldanes LF, Firestone GL (2005). Indole-3-carbinol (I3C) inhibits cyclin-dependent kinase-2 function in human breast cancer cells by regulating the size distribution, associated cyclin E forms, and subcellular localization of the CDK2 protein complex. *The Journal of Biological Chemistry*.

[43] Safe S, Papineni S, Chintharlapalli S (2008). Cancer chemotherapy with indole-3-carbinol, bis(3′-indolyl)methane and synthetic analogs. *Cancer Letters*.

[44] Chen C-Y, Hsu Y-L, Tsai Y-C, Kuo P-L (2008). Kotomolide A arrests cell cycle progression and induces apoptosis through the induction of ATM/p53 and the initiation of mitochondrial system in human non-small cell lung cancer A549 cells. *Food and Chemical Toxicology*.

[45] Firestone GL, Bjeldanes LF (2003). Indole-3-carbinol and 3-3′-diindolylmethane antiproliferative signaling pathways control cell-cycle gene transcription in human breast cancer cells by regulating promoter-Sp1 transcription factor interactions. *The Journal of Nutrition*.

[46] Risau W, Flamme I (1995). Vasculogenesis. *Annual Review of Cell and Developmental Biology*.

[47] Loub WD, Wattenberg LW, Davis DW (1975). Aryl hydrocarbon hydroxylase induction in rat tissues by naturally occurring indoles of cruciferous plants. *Journal of the National Cancer Institute*.

[48] McDanell R, McLean AEM, Hanley AB (1987). Differential induction of mixed-function oxidase (MFO) activity in rat liver and intestine by diets containing processed cabbage: correlation with cabbage levels of glucosinolates and glucosinolate hydrolysis products. *Food and Chemical Toxicology*.

[49] Chen I, McDougal A, Wang F, Safe S (1998). Aryl hydrocarbon receptor-mediated antiestrogenic and antitumorigenic activity of diindolylmethane. *Carcinogenesis*.

[50] Fan S, Meng Q, Saha T, Sarkar FH, Rosen EM (2009). Low concentrations of diindolylmethane, a metabolite of indole-3-carbinol, protect against oxidative stress in a BRCA1-dependent manner. *Cancer Research*.

[51] Srivastava B, Shukla Y (1998). Antitumour promoting activity of indole-3-carbinol in mouse skin carcinogenesis. *Cancer Letters*.

[52] Wattenberg LW, Loub WD (1978). Inhibition of polycyclic aromatic hydrocarbon-induced neoplasia by naturally occurring indoles. *Cancer Research*.

[53] Chen D-Z, Qi M, Auborn KJ, Carter TH (2001). Indole-3-carbinol and diindolylmethane induce apoptosis of human cervical cancer cells and in murine HPV16-transgenic preneoplastic cervical epithelium. *The Journal of Nutrition*.

[54] Davidson B, Goldberg I, Kopolovic J (1997). Angiogenesis in uterine cervical intraepithelial neoplasia and squamous cell carcinoma: An immunohistochemical study. *International Journal of Gynecological Pathology*.

[55] Szakács G, Paterson JK, Ludwig JA, Booth-Genthe C, Gottesman MM (2006). Targeting multidrug resistance in cancer. *Nature Reviews Drug Discovery*.

